# Characterization of intramuscular Isoflupredone acetate in horses: pharmacokinetics and effects on anti-inflammatory mediators and plasma electrolytes

**DOI:** 10.1186/s12917-025-05135-7

**Published:** 2025-11-25

**Authors:** Juliana Sullivan, Jeff Blea, Camilo J. Morales, Daniel S. McKemie, Philip H. Kass, Heather K. Knych

**Affiliations:** 1 K.L. Maddy Equine Analytical Chemistry Laboratory (Pharmacology Section), School of Veterinary Medicine, University of California, Davis, 620 West Health Science Drive, Davis, CA 95616 USA; 2 School of Veterinary Medicine, University of California, Davis, CA USA; 3 Department of Population Health and Reproduction, School of Veterinary Medicine, University of California, Davis, CA USA; 4 Department of Molecular Biosciences, School of Veterinary Medicine, University of California, Davis, CA USA

**Keywords:** Horse, Corticosteroid, Isoflupredone acetate, Detection time, Anti-inflammatory, Hypokalemia

## Abstract

**Background:**

Corticosteroids, such as isoflupredone, are effective anti-inflammatory medications and as such are commonly used to treat inflammation associated with training and injuries in performance horses. While the pharmacokinetics and pharmacodynamics of isoflupredone acetate (IPA) following intra-articular administration to horses has been well described, studies characterizing intramuscular (IM) administration are lacking. The objective of the current study was to describe the pharmacokinetics and anti-inflammatory effects of IPA following IM administration to horses. Twelve horses received a single IM dose of 20 mg IPA, and blood and urine samples were collected starting at 5 min (blood) and 24 h (urine) until 312 h. Concentrations of isoflupredone were determined using liquid chromatography-tandem mass spectrometry, and pharmacokinetic analysis performed. The pharmacodynamic effects of the drug were assessed by measuring endogenous cortisol concentrations and effects on concentrations of inflammatory biomarkers utilizing an ex vivo model of inflammation.

**Results:**

The C_max_, T_max_, and terminal half-life of isoflupredone were 1.55 ± 0.43 ng/mL, 3.50 h (0.16–5.0 h; median and range), and 39.6 ± 22.1 h, respectively. For compartmental modeling, a 1-cmpt model best fit the data. Based on Monte Carlo simulations, for a simulated population of 1000 horses, a detection time of 10 days is recommended for isoflupredone concentrations in 99% of the population to fall below the currently recommended 100 pg/mL regulatory screening limit. Isoflupredone urine concentrations were below the limit of quantitation (0.05 ng/mL) in all horses by 360 h. Significant suppression of endogenous cortisol was observed for 312 h. Stimulation of isoflupredone treated blood with lipopolysaccharide and calcium ionophore resulted in increasing concentrations of several inflammatory biomarkers produced by cyclooxygenase and 15-lipooxygenase, suggesting that the isoflupredone blood concentrations following intramuscular administration may not have been adequate to suppress the activity of these enzymes. A significant decrease in concentration of leukotriene B4 and 5-HETE suggest suppression of 5-lipooxygenase activity by isoflupredone. A single IM administration of IPA resulted in hypokalemia and a significant increase in urinary fractional excretion of potassium.

**Conclusion:**

The prolonged detection time and pharmacologic effects of isoflupredone acetate warrant an extended withdrawal time for IM administration prior to competition in performance horses.

**Supplementary Information:**

The online version contains supplementary material available at 10.1186/s12917-025-05135-7.

## Introduction

Isoflupredone acetate (IPA) is a synthetic glucocorticoid used in veterinary medicine for its anti-inflammatory effects. Although the Food and Drug Administration (FDA) approved veterinary product (Predef 2X, Zoetis, Kalamazoo, MI) has been discontinued, compounded formulations are available in the United States and approved products are available in other countries. Label indications for the previously available FDA approved product and current uses for the compounded product include the treatment of musculoskeletal inflammation, osteoarthritis, and equine asthma, formerly known as recurrent airway obstruction. Since it has anti-inflammatory properties, intra-articular (IA) and intramuscular (IM) administration of IPA to performance horses has been reported according to regulators. As such, its use is restricted prior to competition. Previous studies in horses have described the pharmacokinetics and the duration of select pharmacodynamic effects of IPA when administered IA to horses [1, 2], but there are no reports that describe these characteristics for the IM route of administration in this species.

Exogenous corticosteroid administration suppresses the hypothalamic-pituitary-adrenal axis resulting in a decrease in endogenous cortisol production [3]. Thus, monitoring changes in endogenous cortisol production post corticosteroid administration is commonly used in equine studies assessing the magnitude and duration of effect of intravenous, oral and topical dexamethasone [4–6], IA betamethasone [7], and IM and IA triamcinolone acetonide [8].

As part of their anti-inflammatory effects, corticosteroids modulate the arachidonic acid cascade by binding to the glucocorticoid receptor and acting as a transcription factor for Annexin A1, an inhibitor of the enzyme, Phospholipase A2. This enzyme is responsible for perpetuating the inflammatory cascade through generation of arachidonic acid, a substrate for cyclooxygenase (COX) and lipoxygenase (LOX) enzymes, which produce several eicosanoids that play a role in the inflammatory response. Through production of Annexin A1, corticosteroids inhibit phospholipase A2 [9] and downstream production of these inflammatory mediators. To assess the efficacy of corticosteroids in a pro-inflammatory environment in horses, an ex vivo model has been established in which either lipopolysaccharide (LPS) or calcium ionophore (CI) is added to blood samples collected post drug administration. The cyclooxygenase arm of the arachidonic acid cascade produces 15-hydroxyeicosatetraenoic acid (15-HETE), Prostaglandin E_2_ (PGE_2_) and Prostaglandin F_2 alpha_ **(**PGF_2alpha_) when stimulated by LPS, and concentrations of these products are used as a measure of COX activity. When stimulated with CI, the lipoxygenase arm of the arachidonic acid cascade produces 5-hydroxyeicosatetraenoic acid (5(s)-HETE) and Leukotriene B_4_(LTB4) that are indicators of 5-LOX activity and 15(s)-HETE, an indicator of 15-LOX activity. To assess constitutive COX-1 activity, Thromboxane B2 (TXB2) concentrations are measured in non-stimulated, LPS stimulated, and CI stimulated whole blood. Measuring concentrations of these eicosanoids in an ex vivo model has been effective in understanding the effects of various corticosteroids on the arachidonic acid pathway in horses [6, 10–12].

Another notable effect of isoflupredone (IP), unrelated to its anti-inflammatory effects is hypokalemia. With respect to horses, while the effects of a single administration on electrolyte balance has been reported, hypokalemia has been documented following 14 days of daily IP administration at a dose of 0.03 mg/kg q24h [13]. Given the frequency of administration to performance horses, assessment of the effect of a single administration on electrolyte balance is warranted.

The objectives of this study were to describe the pharmacokinetics and anti-inflammatory effects of a single dose of IM IP as well as its impact on plasma electrolyte concentrations and urinary fractional excretion (FE) 72 h post-administration. We hypothesized that this route of administration will necessitate a prolonged detection time prior to competition, resulting in a long-lived anti-inflammatory effect, and alter plasma potassium concentrations and urinary fractional excretion.

## Methods

### Animals

Twelve healthy Thoroughbred horses (8 geldings, 4 mares; 4–7 years of age; weight: 556.5 ± 58.4 kg) were enrolled. The number of horses was selected based on commonly used sample sizes for studies conducted for the purpose of establishing regulatory recommendations for performance horses in the United States. All horses belonged to the exercised research herd at the School of Veterinary Medicine, University of California, Davis. Horses were exercised five days a week according to a standardized exercise regimen: three days on a mechanical walker (5 min walk, 15 min trot, 5 min walk; Centaur Horse Walkers Inc., Mira Loma, CA), and two days on a high-speed treadmill (3 min at 1.9 m/s, 5 min at 4 m/s, 2 min at 7 m/s, 1 min at 9 m/s, and 5 min walk at 1.9 m/s; Mustang 2200, Graber AG, Switzerland). This protocol continued throughout the study, except on the day of drug administration.

Horses were not administered any medications for at least four weeks prior to enrollment and were confirmed healthy based on physical examination, complete blood count, and serum biochemistry. Blood analyses were conducted by the Clinical Pathology Laboratory at the William R. Pritchard Veterinary Medical Teaching Hospital using standard protocols. The study was approved by the Institutional Animal Care and Use Committee of the University of California, Davis.

### Drug administration

Immediately before drug administration, a 14-gauge catheter was placed in one external jugular vein for sample collection, and body weight was recorded. As there is currently no commercially available formulation available, a compounded formulation was utilized. Since a compounded product was used, the concentration was determined by liquid chromatography-tandem mass spectrometry (LC-MS/MS) as described below, prior to administration to ensure the labeled concentration. Each animal received a single IM administration of 20 mg of IPA into the cervical serratus ventralis muscle. The IPA dose was selected based on the label dose for IM administration to horses for the recently discontinued FDA approved product (5–20 mg). An informal survey of racetrack practitioners indicates this dose is still commonly used in clinical practice for compounded products.

### Sample collection

For drug concentration determination, blood samples were collected at baseline (0 min) and at 5, 10, 15, 30, and 45 min, then at 1, 2, 3, 4, 5, 6, 8, 12, 18, 24, 30, 36, 48, 60-, 72-, 96-, and 120-hours post-administration. After the 18-hour timepoint, catheters were removed, and subsequent samples were collected via direct venipuncture. The 120-hour samples were analyzed immediately via LC-MS/MS. Isoflupredone concentrations were above the limit of quantitation (LOQ) and thus additional samples were collected on days 7, 8, 9, 10, and 13 until levels dropped below the limit of detection (LOD). Blood was collected into EDTA tubes (Kendall/Tyco Healthcare, Mansfield, MA), centrifuged at 3,000 × g, and plasma was stored at −20 °C in cryovials until analysis (within two weeks of final collection).

Blood samples for determination of cortisol concentrations were collected as described for IP concentrations starting at 30 min post ISO administration.

Eicosanoid concentrations (5-HETE, LTB,15-HETE, TXB2, PGE2, and PGF2α) were measured in whole blood collected at baseline (0 min) and at 1, 2, 3, 4, 5, 6, 8, 12, 24, 30, 48, 60, 72, 96, and 120 h and on days 7, 8, 9, 10, and 13. At each time point, one sample was collected into a non-heparinized tube, incubated at 37 °C for 1 h, centrifuged, and the resulting serum was stored at − 20 °C until analysis for determination of TXB2 (unstimulated sample). A second blood sample was collected into an EDTA tube and divided into three 3 mL aliquots for ex vivo stimulation. One aliquot was treated with calcium ionophore (10 µM in 2% methanol) and incubated for 2 h at 37 °C; a second was stimulated with lipopolysaccharide (50 µg/mL in water) and incubated for 24 h at 37 °C; the third served as a vehicle control and was treated with 2% methanol and incubated for 2 h at 37 °C. Plasma from all aliquots was harvested by centrifugation and stored at − 20 °C until determination of eicosanoid concentrations by LC-MS/MS.

Urine samples were collected by free catch at 24, 48-, 72-, 96-, and 120-hours post-administration. As with plasma, the 120-hour sample was analyzed immediately. As drug was still detectable, additional samples were obtained on days 7, 8, 9, 10 and 13. All urine samples were stored at −20 °C until analyzed by LC-MS/MS.

### Determination of Isoflupredone concentrations

#### Plasma sample analysis

The concentration of IP in plasma samples was determined using a previously validated and published LC-MS/MS method [2]. Calibration curves and negative control samples were prepared fresh for each quantitative assay. Quality control samples (equine plasma fortified with ISO at three concentrations within the standard curve) were included with each sample set as a check of accuracy.

#### Urine sample analysis

The concentration of ISO in urine samples was determined using a previously validated and published LC-MS/MS method [2]. Samples for the calibration curve and negative control samples were prepared fresh for each assay. Quality control samples (equine urine fortified with analyte at three concentrations within the standard curve) were included with each sample set as a check of accuracy.

### Cortisol concentration determination

Cortisol concentrations in plasma were quantified using a LC-MS/MS method that was previously validated and published [12]. Calibration curves and negative controls were freshly prepared for each assay, with QC samples (plasma fortified at three concentrations within the calibration range) included for accuracy verification.

### Eicosanoid concentration determination

Eicosanoids (5-HETE, LTB4, 15-HETE, TXB2, PGE2, PGF2α) were quantified using a validated LC-MS/MS method [12]. Quality control samples, consisting of equine blood fortified with known analyte concentrations at three points within the standard curve, were included in each batch to ensure accuracy.

### Pharmacokinetic analysis

Non-compartmental analysis (NCA) was performed using Phoenix WinNonlin (v8.2, Certara, Princeton, NJ) to generate initial pharmacokinetic estimates. Concentration-time data were subsequently analyzed using nonlinear mixed-effects (NLME) population modeling in Phoenix NLME. A first-order conditional estimation with interaction (FOCE-ELS) algorithm was employed to evaluate one- and two- compartment models incorporating various residual error structures (additive, multiplicative, add + mult, Poisson, and mixed). Model selection was based on visual inspection of observed vs. predicted concentration plots, residual diagnostics, Akaike Information Criterion (AIC), and the coefficient of variation (%CV) of parameter estimates.

### Monte Carlo simulation

Monte Carlo simulations were performed to determine the time at which 99% of the thoroughbreds in a simulated population would fall below the Racing Medication and Testing Consortium (RMTC) screening limit (SL) for IP of 100 pg/mL. To accomplish this, the sample of 12 thoroughbreds was simulated 1000 times from time 0 to 408 h in 3-hour increments. A 95% confidence level was used. At each simulated sample time post-dosing, the 50th and 99th percentile were computed and then the times post-dosing were plotted vs. the 50th and 99th percentiles to determine the time at which both fell below the 100 pg/mL SL.

### Fractional excretion determination

Plasma and urine concentrations of potassium, sodium, chloride, and phosphorus were measured at baseline and 72 h post-administration using ion-selective electrodes, as validated for equine samples by the Clinical Pathology Laboratory at the William R. Pritchard Veterinary Medical Teaching Hospital using standard protocols. Fractional excretion (FE) of each electrolyte (X) was calculated using the following formula:

$$FX_x=\left[\left(Urine_x/Plasa_x\right)\div\left(Urine_{creatinine}/Plasma_{creatinine}\right)\right]\times100$$

### Statistical analysis

Statistical analyses were performed using Stata/BE 17.0 (StataCorp, TX, USA) to evaluate differences in hydrocortisone and eicosanoid concentrations between baseline and post-administration time points. For plasma electrolytes and percent fractional excretion, baseline (pre-drug administration) values were compared to values at 72 h post IPA administration. For all analyses, a mixed-effects ANOVA was used, with horse as a random effect and time as a fixed effect. Post hoc comparisons were adjusted using the Bonferroni method to maintain a nominal significance level of 0.05.

## Results

Isoflupredone acetate was well tolerated with no reactions noted at the site of injection in any horses. The concentration of the compounded dosing formulation was 1.99 mg/mL (99.5% of the nominal (2 mg/mL) concentration).

The instrument response was linear with correlation coefficients of 0.99 or better. Accuracy and precision are reported as percent nominal concentration and percent relative standard deviation (SD) respectively and were all within ± 15% of the nominal value (Supplementary Tables 1 and 2). The LOQ and LOD for all analytes are listed in Table 1.

**Table 1:** Limit of quantitation (LOQ) and limit of detection (LOD) for isoflupredone, cortisol, and eicosanoids

Analyte		LOQ	LOD
Isoflupredone			
	Plasma	0.01ng/mL	0.005 ng/mL
	Urine	0.05 ng/mL	0.0025 ng/mL
Cortisol		1.0 ng/mL	0.5 ng/mL
TXB2		0.1 ng/mL	0.05 ng/mL
PGE2		0.05 ng/mL	0.025 ng/mL
PGF2alpha		0.1 ng/mL	0.05 ng/mL
LTB4		0.05 ng/mL	0.02 ng/mL
15-HETE		0.1 ng/mL	0.05 ng/mL
5-HETE		0.25 ng/mL	0.1 ng/mL

**Fig. 1:**
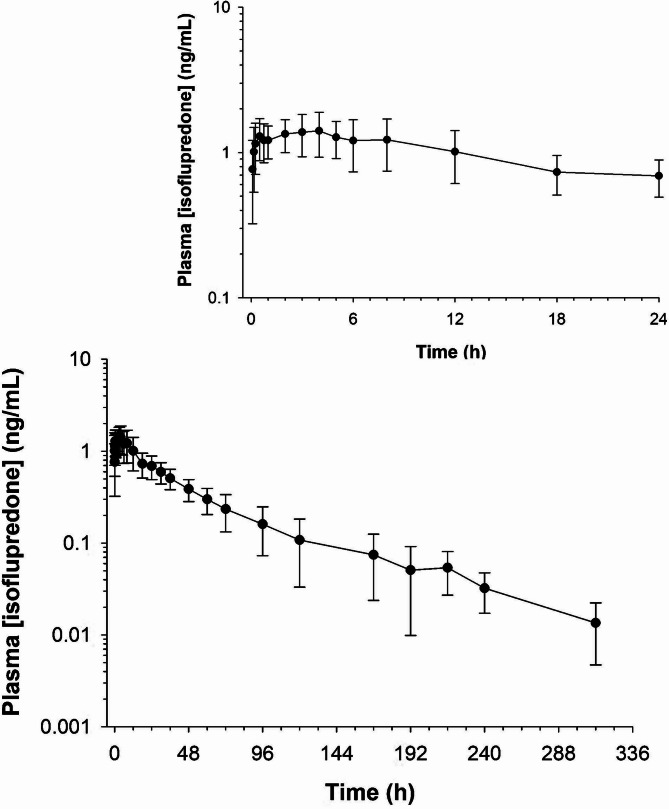
Plasma isoflupredone concentrations (mean + SD) following a single intramuscular dose of 20 mg isoflupredone acetate to 12 horses. Inset shows concentrations over the first 24 hours

Plasma concentrations of IP over time are depicted in Fig. 1. Concentrations were below the RMTC SL (0.1 ng/mL) at 216 h in all horses. Isoflupredone concentrations in plasma were below the LOQ (0.01 ng/mL) in 10/12 horses by 312 h (the last time point collected). Two horses had concentrations of 0.02 ng/mL at the final time point sampled. The results of non-compartmental analysis are listed in Table 2. The maximum concentration (C_max_) ranged from 0.95 to 2.45 ng/mL and time of maximum concentration (T_max_) from 0.16 to 5.0 h. The terminal half-life ranged from 13.6 to 74.1 h. For compartmental modeling, a one-compartment population model with a multiplicative residual error structure gave the best fit to the plasma concentration-time data. Pharmacokinetic parameters (estimate and % coefficient of variation for the fixed and random effects) generated from the compartmental model are listed in Table 3.

**Table 2:** Pharmacokinetic parameters for Isoflupredone following intramuscular administration of 20 mg of Isoflupredone acetate to 12 horses. Parameters were generated using non-compartmental analysis

Parameter	Mean ± SD	Median	Range
C_max_ (ng/mL)	1.55 ± 0.43	1.49	0.95–2.45
T_max_ (h)	2.81 ± 1.78	3.50	0.16–5.0
AUC_inf_ (ng hr/mL)	58.8 ± 9.85	58.3	36.9–72.2
AUC extrap (%)	2.39 ± 2.54	1.31	0.51–8.84
Terminal HL (h)	39.6 ± 22.1	34.7	13.6–74.1

*C*_*max*_ maximum plasma concentration, *T*_*max*_ time of maximum concentration, *AUCinf* area under the curve to infinity, *AUC extrap* percentage of AUCinf that is extrapolated

**Table 3:** Model typical values (tv) for Isoflupredone following a single intramuscular dose of 20 mg of Isoflupredone acetate to 12 horses

Parameter	Estimate	CV(%)
tvKa (1/h)	8.34	22.7
tvV/F (L/kg)	27.9	10.2
tvCl/F (mL/min/kg)	10.1	6.17
stdev0	0.187	10.1
Ke (1/h)	0.022	14.6
HL Ke	32.0	14.6
Between subject variability		
Ka	0.596	90.3
V	0.123	36.2
Cl	0.049	22.3

*tvKa* rate of absorption, *tvV* the value of the central compartment, *tvCl* the clearance of drug from plasma, *Ke* elimination rate constant, *HL Ke HL* half-life, stdev0, the estimated residual standard deviation for plasma data. CV(%), coefficient of variation

**Fig. 2:**
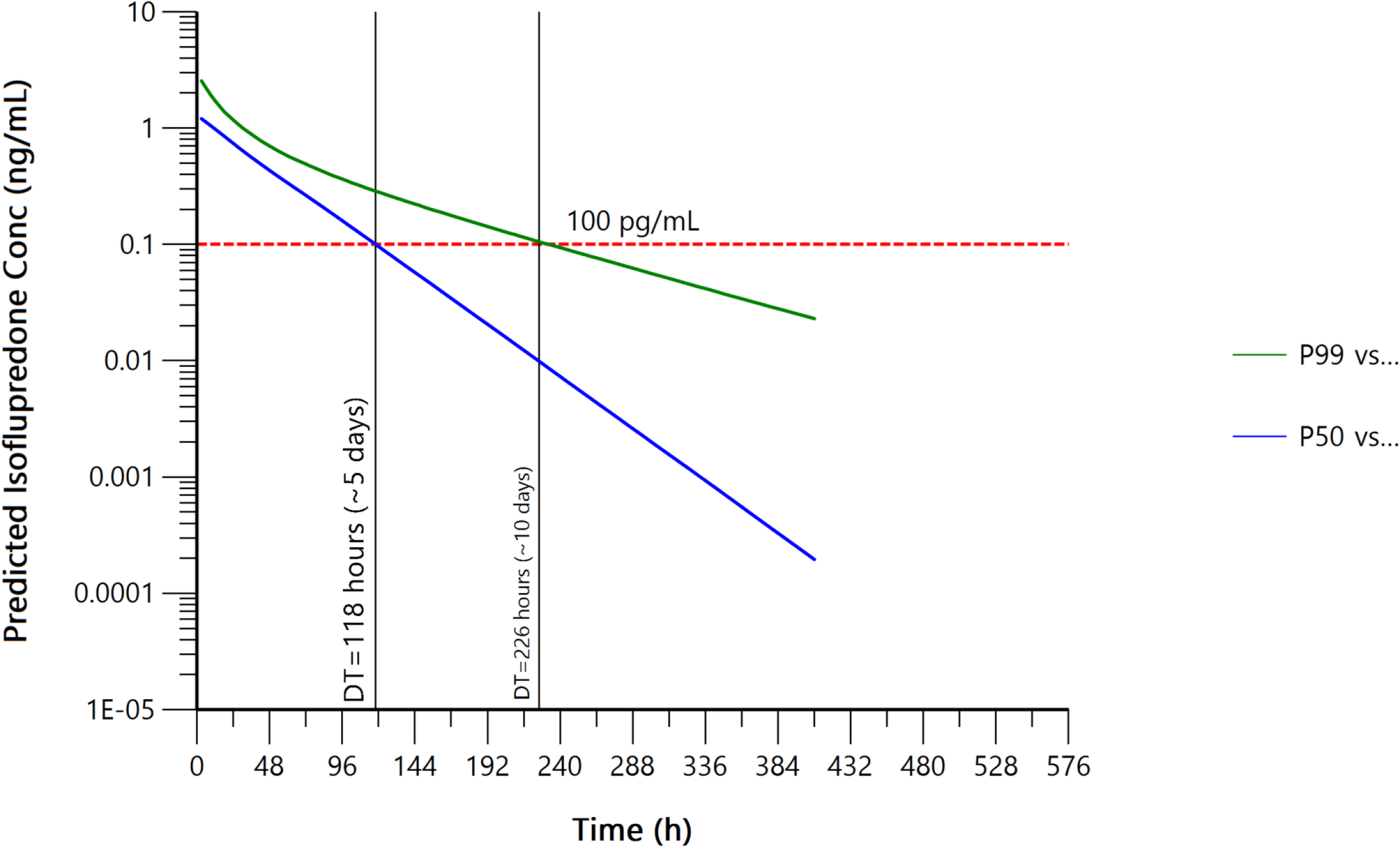
Plot of the 50% and 99% quantiles for a hypothetical horse population (1000 Thoroughbred horses) obtained via Monte Carlo simulation for the screening limit of 100 pg/mL (0.1 ng/mL) for isoflupredone. The times of intersection of the predicted concentration curves with the screening limit line represent the detection time for 50% and 99% of the hypothetical population

Based on the Monte Carlo simulations, the time at which 50% and 99% of the simulated population would fall below the RMTC recommended SL for IP of 100 pg/mL (0.1 ng/mL) was approximately 118 h (~ 5 days) and 226 h (~ 10 days), respectively (Fig. 2).

Urine IP concentrations are listed in Table 4. Concentrations were below the limit of quantitation (0.05 ng/mL) in all horses by 360 h post administration (the last time point measured).

**Table 4:** Isoflupredone urine concentrations following a single intramuscular administration of 20 mg of Isoflupredone acetate to 12 horses

Time (h)	Urine concentrations (ng/mL)	Number of horses > LOQ
24	16.3 ± 11.9	12/12
48	11.9 ± 4.47	12/12
72	6.27 ± 2.94	12/12
96	4.33 ± 2.25	12/12
120	3.20 ± 2.55	12/12
168	2.14 ± 1.42	11/12
192	1.41 ± 1.32	11/12
216	1.04 ± 0.80	10/12
240	0.71 ± 0.63	9/12
312	0.49 ± 0.13	7/12
336	0.15 ± 0.09	2/12
360	ND	0/12

*LOQ* limit of quantitation (0.05 ng/mL), *ND* not detected

**Fig. 3:**
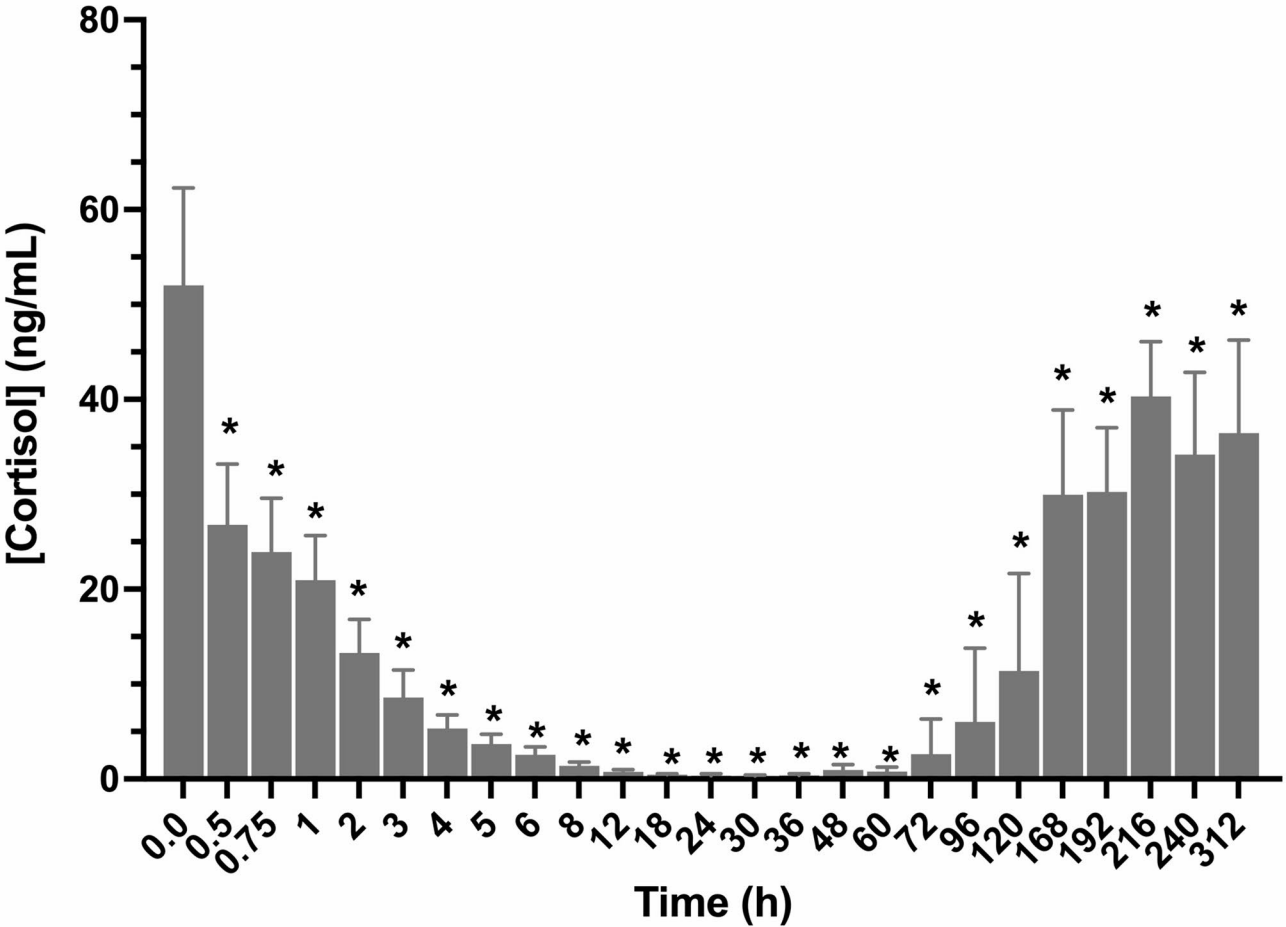
Plasma cortisol concentrations following a single intramuscular administration of 20 mg of isoflupredone acetate to 12 horses. *, indicate a statistically significant difference, relative to baseline

Cortisol concentrations (mean ± SD) are depicted in Fig. 3. Baseline cortisol concentrations (prior to IPA administration; collected at approximately 7 a.m.) were 52.0 ± 10.3.7 ng/mL (mean ± SD). Cortisol concentrations were significantly reduced (*p* < 0.05), relative to baseline, starting at 30 min and through the duration of sample collection (312 h). The corresponding plasma IP concentration at 312 h ranged from non-detectable to 0.01 ng/mL. The lowest cortisol concentration was observed at 36 h post administration.

In the ex vivo assay, methanol stimulated whole blood (control) did not result in measurable concentrations of eicosanoids, except for TXB2.

**Fig. 4:**
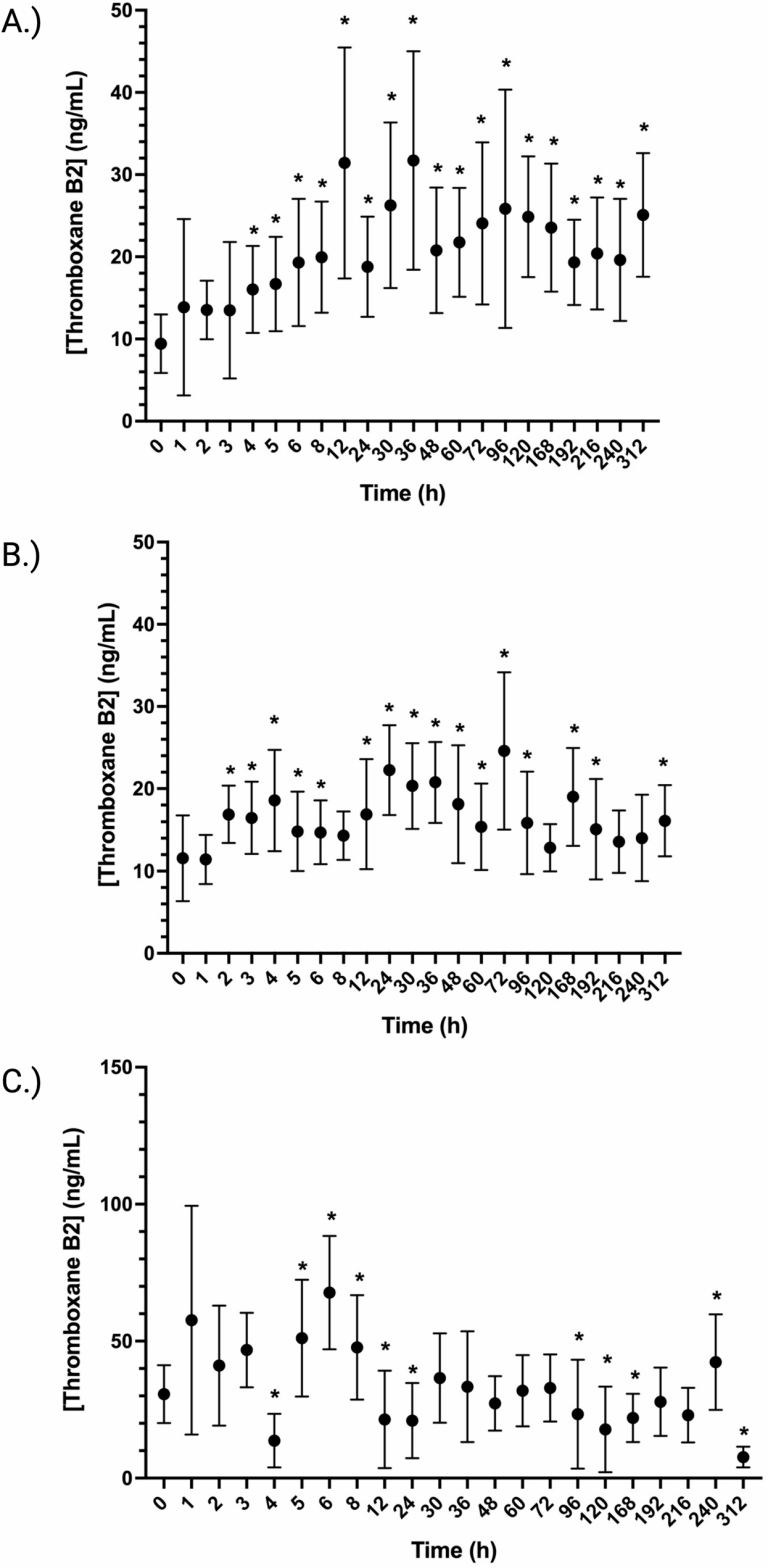
Effects of a single administration of 20 mg of isoflupredone acetate on Thromboxane B2 (TXB2) in serum samples (**A**) lipopolysaccharide treated whole blood (**B**) or calcium ionophore treated whole blood (**C**). *, indicates concentrations that are significantly (*p*<0.05) different from baseline (prior to drug administration)

In non-stimulated serum samples, concentrations of TXB2 were significantly increased (*p* < 0.05) starting at 4 h post IPA administration. Concentrations remained increased through the duration of the study collection timepoints (312 h) (Fig. 4A). Stimulation of blood samples with LPS, resulted in an increase in TXB2 concentrations from 3 to 6, 12–96, 168-, 192- and 312-hours post administration (Fig. 4B). Significant increases, relative to baseline in TXB2 in CI stimulated blood occurred from 5 to 8 and at 240 h and significant decreases were observed at 4, 12-, 24-, 96–168-, and 312-hours post IPA administration.

**Fig. 5:**
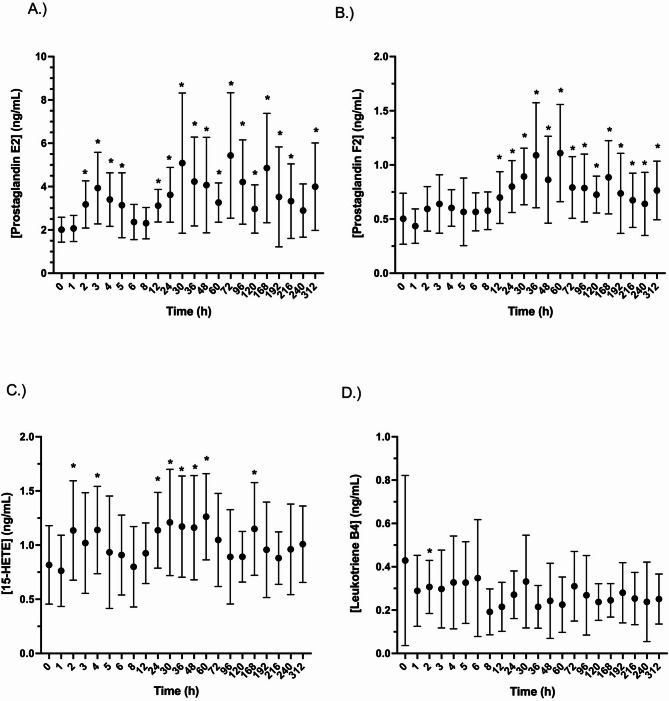
Effects of a single administration of 20 mg of isoflupredone acetate on A). Prostanglandin E2, B). Prostanglandin F2, C). 15-HETE, and D). Leukotriene B4 production in lipopolysaccharide treated equine whole blood at various times post drug administration. *, indicates concentrations that are significantly (*p*<0.05) different from baseline (prior to drug administration)

Following LPS stimulation, concentrations of PGE_2_ were significantly increased (*p* < 0.05) from 2 to 5, 12–216, and at 312 h post administration (Fig. 5A). Concentrations of PGF2 alpha were significantly increased (*p* < 0.05) from 1 to 8 h and significantly increased from 12 to 312 h in LPS (Fig. 5B) stimulated blood. Following LPS stimulation, concentrations of 15-HETE were significantly increased at 2- and 8-hours post administration and again from 24 to 60 and 168 h (Fig. 5C). Except for at 2 h, concentrations of LTB4 in LPS stimulated blood were unchanged post IPA administration (Fig. 5D).

**Fig. 6:**
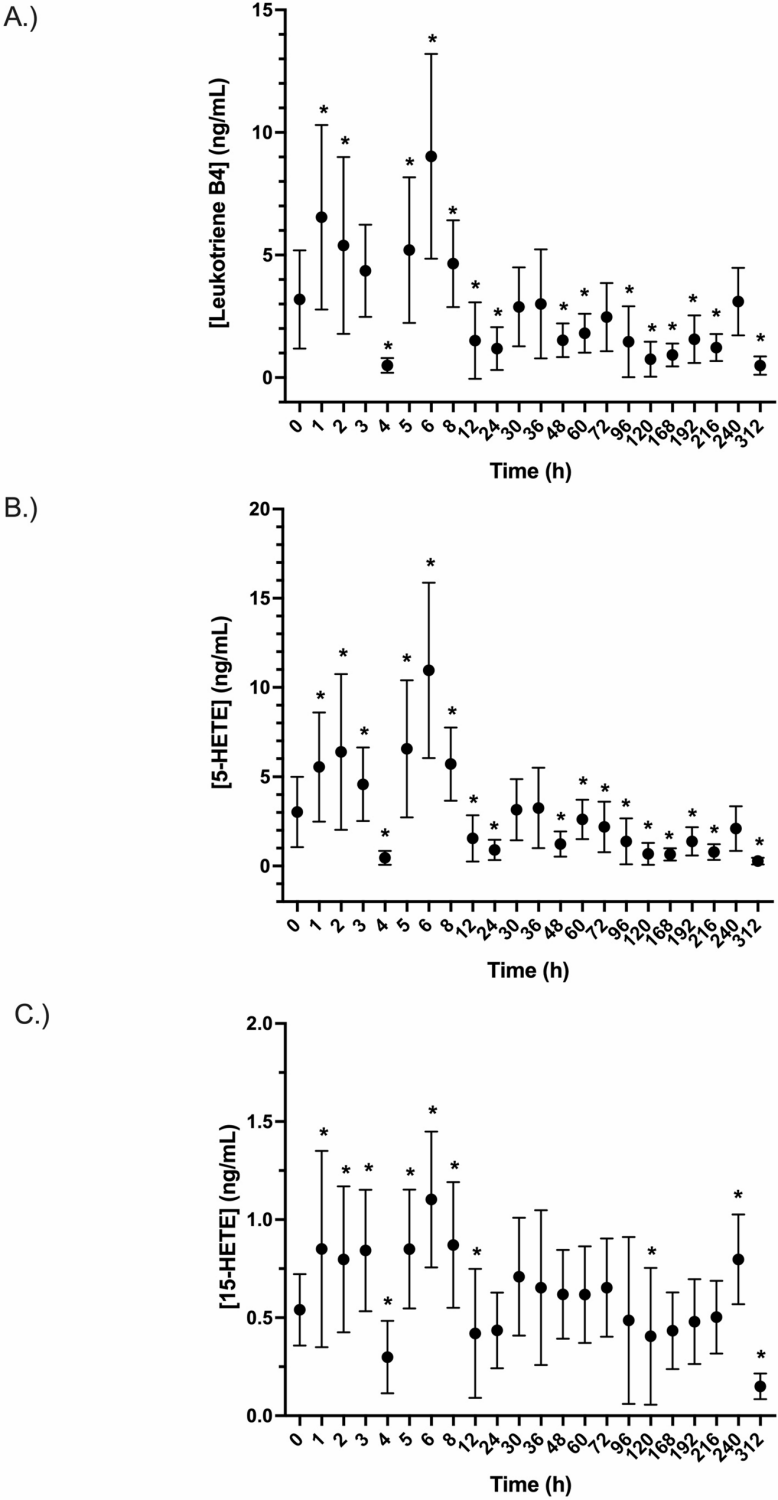
Effects of a single administration of 20 mg of isoflupredone acetate on A). Leukotriene B4, B). 5-HETE, and C). 15-HETE production in calcium ionophore treated equine whole blood at various times post drug administration. *, indicates concentrations that are significantly (*p*<0.05) different from baseline (prior to drug administration)

In CI stimulated blood, except for the 4-hour time point, both LTB4 and 5-HETE concentrations were significantly (*p* < 0.05) increased for the first 8 h post administration. Starting with the 12 h time point, concentrations of both decreased significantly, relative to baseline (Fig. 6A and B). Concentrations of 15- HETE were significantly increased, relative to baseline for the first 8 h post administration (except for the 4-hour time point) (Fig. 6B and C). A significant decrease in 15-HETE was noted at 12 h, however levels thereafter were not significantly different from baseline.

**Fig. 7:**
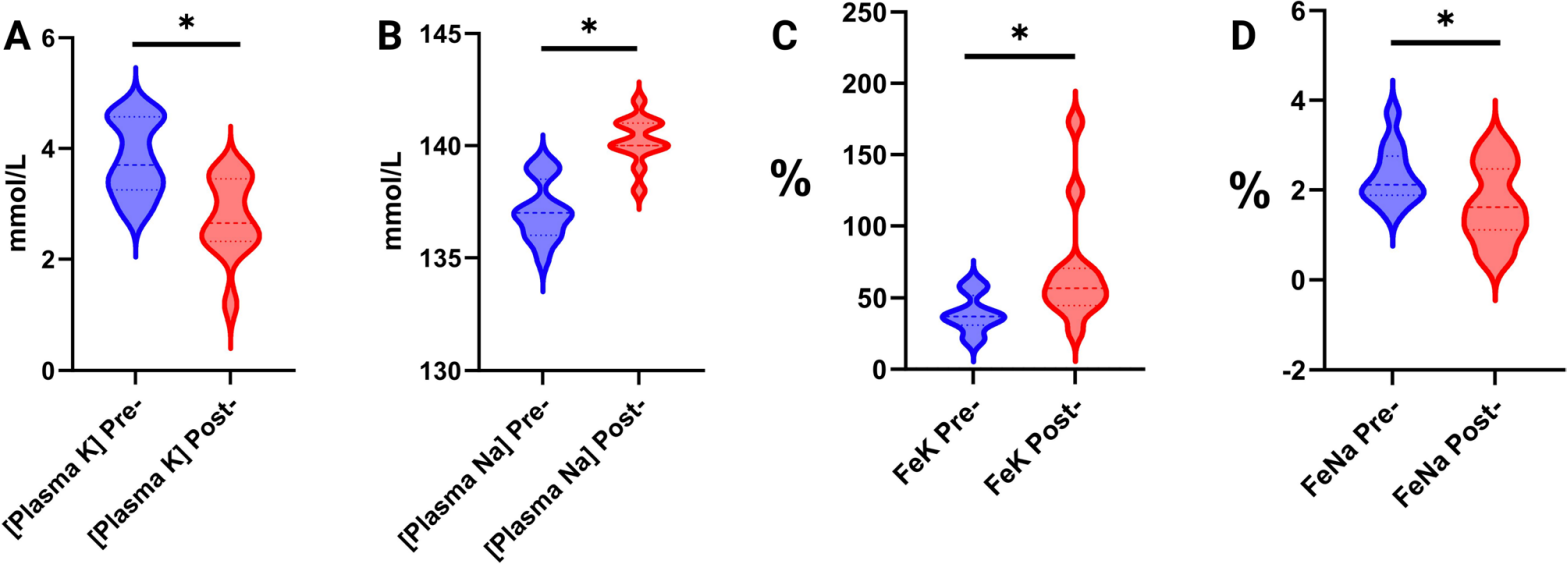
Violin plots of: **A**) plasma potassium; **B**) plasma sodium; **C**) urinary fractional excretion of potassium; and **D**) urinary fractional excretion of sodium, before and after intramuscular administration of 20 mg of isoflupredone to 12 horses. * indicates a significant difference; *p* < 0.05

Plasma potassium concentrations measured at 72 h post IP administration were significantly decreased (2.7 ± 0.71 mmol/L) compared to baseline (3.8 ± 0.67 mmol/L; *p* < 0.001; Fig. 7A). Plasma sodium significantly increased post-administration [140 mmol/L (138–142)] compared to pre-administration [137 mmol/L (135–139); *p* < 0.001] (Fig. 7B). Urinary fractional excretion (FE) of potassium increased from 39% (20–61) to 68.9% (27–73; *p* = 0.011; Fig. 7C), while sodium FE decreased from 2.3% (1.47–3.72) to 1.61% (0.51–3.03; *p* = 0.01; Fig. 7D). There were no significant differences in plasma electrolyte concentrations or in the fractional excretion of chloride and calcium (Table 5).

**Table 5:** Electrolyte plasma concentrations and urinary fractional excretion (%FE) before and after intramuscular administration of 20 mg of Isoflupredone to 12 horses

Analyte	[Plasma Electrolyte]		*p*-value	% FE		*p*-value
	Pre-	Post-		Pre-	Post-	
Potassium – mmol/L	3.8 ± 0.71	2.7 ± 0.67	*p* < 0.001	39 (20–61)	69 (27–73)	*p* = 0.011
Sodium – mmol/L	137 (135–139)	140 (138–142)	*p* < 0.001	2.3 (1.47–3.72)	1.61 (0.51–3.03)	*p* = 0.011
Chloride – mmol/L	100.1 (97–102)	99.9 (99–101)	*p* = 0.613	3.7 (1.4–20)	2.2 (1.4–3.2)	*p* = 0.279
Calcium – mg/dL	11.6 (11–12)	11.8 (11–12)	*p* = 0.212	4.7 (0.7–21)	12.1 (1.2–45)	*p* = 0.064
Phosphorus – mg/dL	3.3 ± 0.66	3.3 ± 0.71	*p* = 0.975	0.02 (0.01–0.04)	0.02 (0.008–0.03)	*p* = 0.039

*p* value represents comparison between pre and post values for each electrolyte and the %FE

## Discussion

The current study describes plasma and urine concentrations, pharmacokinetics and anti-inflammatory effects of IPA following IM administration to horses, with the goal of providing guidance to veterinarians for establishing withdrawal time recommendations.

The current RMTC regulatory threshold for IP is 100 pg/mL (0.1 ng/mL) with a corresponding withdrawal time of 7 days for a 20 mg intra-articular or 10 mg subcutaneous dose. While a specific withdrawal time recommendation is not made for IM administration, the RMTC does caution that administration of IP by this route will result in concentrations more than the threshold for an extended period [14]. Based on an RMTC monograph, serum IP concentrations are still above the threshold in some horses (16–110 pg/mL) following intramuscular administration of 20 mg, at 168 h (last time point reported) post administration [15]. In the current study, concentrations fell below the threshold in all horses at 216 h following IM administration of the same dose. The prolonged detection time following IM administration is further supported by Monte Carlo simulations. Pharmacokinetic data from the 12 horses administered IPA was used to simulate a herd of 1000 Thoroughbred horses. Based on these simulations, it would take 5 days for 50% of the simulated population to fall below the RMTC SL of 100 pg/mL and 10 days for 99% of the population to do so.

While the pharmacokinetics of IPA following intra-articular administration have been described, to the best of the authors’ knowledge there are no pharmacokinetic reports following IM administration to horses. The C_max_ reported here following IM administration of 20 mg was 1.55 ng/mL (mean) which is similar to reports following intra-articular administration of 8 mg in a single joint (1.53 ng/mL). The mean T_max_ was also similar between routes of administration (3.34 h for intra-articular vs. 2.81 h for IM).

The terminal half-life for IPA in the current study was long (38.7 ± 20.2 h). This is consistent with other reports describing the pharmacokinetics of IM and IA corticosteroid ester formulations in horses. In other studies, investigators have postulated that this may be due to flip-flop kinetics, whereby the rate of absorption is slower than the rate of elimination. Thus, the long terminal phase of the concentration curve is largely a result of the slow rate of absorption as opposed to elimination. Although intravenous administration of IP was not conducted in the current study and to the authors’ knowledge there are no published reports describing the pharmacokinetics of intravenous IP, flip flop kinetics may explain the long terminal half-life in the current study. The terminal half-life reported here (38.7 h) was more prolonged compared to intra-articular administration (24.2 h) [2]. This supports the need for a longer withdrawal time following IM versus intra-articular administration of IPA and is in agreement with reports describing the pharmacokinetics of triamcinolone acetonide following administration by these two routes [16].

In the current study, endogenous cortisol was suppressed for at least 312 h (13 days; last time point measured) post administration of IPA. Following IM administration of 0.03 mg/kg IPA daily for 14 days to horses, investigators reported that cortisol concentrations were still suppressed 21 days (last time point assessed) after the last dose [13], in agreement with the prolonged suppression observed in the current study. This long duration of cortisol suppression has also been reported following administration of several other corticosteroids to horses [6, 7, 12, 17].

To assess the effects of IP on inflammatory mediators, the concentrations of products of the arachidonic acid pathway were measured in both non-stimulated and LPS and CI stimulated blood. Thromboxane B2 concentrations in unstimulated blood samples and those stimulated with CI were increased or remained unchanged following drug administration, suggesting that IP either does not affect the constitutively expressed form of COX-1 or that blood concentrations of IP were too low to suppress the activity of this enzyme. The findings in the current study agree with previous reports utilizing these assessments with other corticosteroids administered to horses. As with the unstimulated and CI stimulated blood, TXB2 concentrations in LPS stimulated samples were also significantly increased following IPA administration. Similarly, increases in concentration of other COX-2 products (PGE2, PGF2 alpha and 15-HETE) were increased. This is in contrast to what has been reported for other corticosteroids following administration to horses and using this same model, whereby deceased concentrations of COX-2 products was observed [6, 10, 12]. Although it would be necessary to study additional doses, the findings presented here suggest that, as theorized for COX-1 above, blood concentrations of IP may not have been sufficient to inhibit COX-2 following stimulation of blood with LPS.

In the ex vivo model utilized here, addition of CI to blood stimulates 5-LO, increasing concentrations of 5-HETE and LTB and 15-LO increasing synthesis of 15-HETE. Concentrations of LTB4, 5-HETE, and 15-HETE all increase for the first several hours post IPA administration. However, starting with the 12-hour sample, both LTB4 and 5-HETE concentrations were statistically decreased, relative to baseline. Although observation of the effect is delayed, this finding supports inhibition of 5-LO by IP. Concentrations of 15-HETE decreased significantly relative to baseline at only one time point (8 h), suggesting that 15-LO was only minimally affected by IP, at least at the blood concentrations achieved in the current study.

Several factors must be taken into consideration when interpreting the findings of the current study. Firstly, as there is no commercially available FDA approved product, a compounded formulation was used. The specific product administered in the current was analyzed prior to administration and the concentration was consistent with the label concentration. However, this may not apply to every compounded product, even the same formulation purchased from the same compounding pharmacy, as compounded products are not necessarily subject to the same rigorous quality control standards as FDA-approved products. A potential limitation of the current study is the use of an ex vivo model to study the effects of IP on inflammatory mediators. Although the model used in the current study is well-established and has been used to assess the effects of drugs on inflammatory mediators in both humans and veterinary species, it is important to note that ex vivo and in vitro models are not necessarily fully reflective of in vivo conditions. Additionally, results of the inflammatory mediator analysis suggest a lack of effect of IP on COX enzymes and perhaps a minimal effect on 15-LO. However, it is important to note that only a single dose was studied. It would therefore be prudent to further evaluate the concentration-effect relationship for IP, and more specifically determine the IC50 or IC90 for inhibition of COX and LOX enzymes,

Hypokalemia and increased urinary fractional excretion of potassium have been reported in horses and cattle following IM administration of IPA [13, 18]. In the current study, a single intramuscular administration of 20 mg IPA significantly decreased plasma potassium and increased plasma sodium concentrations, consistent with the observed changes in their respective FEs 72 h post administration. Changes in potassium have been attributed to the mineralocorticoid effect of IPA [18] and changes in serum sodium and the corresponding decrease in urinary fractional excretion are likely attributable to the kidneys efforts to maintain electroneutrality [19].

Hypokalemia can result in undesirable affects. Fatal arrhythmias have been associated with hypokalemia in humans [20] and in horses, atrial fibrillation has been linked to hypokalemia [21]. With respect to IPA, atrial fibrillation was reported in a cow that received two doses [18]. Although, in the present study, no clinical signs of hypokalemia were observed, further study would be prudent, including continuous ECG monitoring, to assess the cardiac effects of IPA in horses, especially given its frequent administration to horses undergoing intensive exercise.

## Conclusion

This study describes the pharmacokinetics and pharmacodynamics of IM IPA in horses. The long detection time of IP following IM administration of IPA warrants an extended withdrawal time prior to competition to prevent an adverse analytical finding. Furthermore, the prolonged suppression of endogenous cortisol production and 5-LO activity suggests an extended pharmacologic effect. Based on effects on plasma potassium concentrations and changes in urinary fractional excretion, additional study of the cardiovascular effects of IM administration of IPA are warranted. Additionally, as corticosteroids, such as IP, have the potential to induce endocrinopathic laminitis, it may be worthwhile to study the effects of higher dosages and repeat dosing on glucose and insulin over time.

## Supplementary Information

Supplementary Material 1.

## Data Availability

The datasets used and/or analyzed during the current study are available from the corresponding author on reasonable request.

## Abbreviations

5-HETE: 5-hydroxyeicosatetraenoic acid
5-LO: 5-liopooxygenase
15-HETE: 15-hydroxyeicosatetraenoic acid
15-LO: 15-lipooxygenase
AIC: , Akaike Information Criterion
CI: calcium ionophore
C_max_: maximum concentration
COX: cyclooxygenase
FDA: Food and Drug Administration
FE: fractional excretion
IA: intra-articular
IM: intramuscular
IP: isoflupredone
IPA: isoflupredone acetate
LC-MS/MS: liquid chromatography mass spectrometry/mass spectrometry
LOD: limit of detection
LOX: lipoxygenase
LOQ: limit of quantitation
LPS: lipopolysaccharide
LTB4: Leukotriene B_4_
NCA: non-compartmental analysis
NLME: nonlinear mixed-effects
PGE_2_: Prostaglandin E_2_
PGF_2alpha_: Prostaglandin F_2 alpha_
RMTC: Racing Medication and Testing Consortium
SL: screening limit
T_max_: time of maximum concentration
TXB2: thromboxane B2
