# Supplementary material for: Characterization of intramuscular Isoflupredone acetate in horses: pharmacokinetics and effects on anti-inflammatory mediators and plasma electrolytes
Source: BMC Vet Res. 2025 Nov 25;21:685. doi: 10.1186/s12917-025-05135-7 (PMC12649081; doi:10.1186/s12917-025-05135-7)
Supplement: Supplementary file 1 — Supplementary Material 1. [file 12917_2025_5135_MOESM1_ESM.docx]

**Supplementary Information:**

**Supplementary Table 1.** Accuracy and precision values for LC-MS/MS analysis of isoflupredone and cortisol in equine biological matrices. Values represent the average of 6 replicates.

| Analyte | Matrix | Concentration  (ng/mL) | Intra-day accuracy (% nominal concentration) | Intra-day precision  (% relative SD) |
| --- | --- | --- | --- | --- |
| Isoflupredone |  |  |  |  |
|  | Plasma |  |  |  |
|  |  | 0.15 | 91.0 | 4.0 |
|  |  | 2.0 | 97.0 | 8.0 |
|  |  | 9.0 | 92.0 | 8.0 |
|  |  |  |  |  |
|  | Urine |  |  |  |
|  |  | 0.3 | 103 | 5.0 |
|  |  | 2.0 | 106 | 4.0 |
|  |  | 9.0 | 109 | 5.0 |
| Cortisol |  |  |  |  |
|  | Plasma |  |  |  |
|  |  | 20.0 | 89.0% | 13.0 |
|  |  | 80.0 | 107% | 8.0 |

**Supplementary Table 2.** Accuracy and precision values for LC-MS/MS analysis of various eicosanoids in equine blood.

| Analyte | Concentration  (ng/mL) | Intra-day accuracy (% nominal concentration) | Intra-day precision  (% relative SD) |
| --- | --- | --- | --- |
| TXB2 |  |  |  |
|  | 0.3 | 97.0 | 8.0 |
|  | 4.0 | 100 | 3.0 |
|  | 40.0 | 104 | 4.0 |
| PGE2 |  |  |  |
|  | 0.3 | 102 | 12.0 |
|  | 4.0 | 101 | 14.0 |
|  | 40.0 | 107 | 4.0 |
| PGF2alpha |  |  |  |
|  | 0.3 | 105 | 4.0 |
|  | 4.0 | 99.0 | 4.0 |
|  | 40.0 | 101 | 4.0 |
| LTB4 |  |  |  |
|  | 0.3 | 103 | 5.0 |
|  | 4.0 | 100 | 3.0 |
|  | 40.0 | 102 | 4.0 |
| 15-HETE |  |  |  |
|  | 0.3 | 102 | 9.0 |
|  | 4.0 | 99.0 | 6.0 |
|  | 40.0 | 101 | 4.0 |
| 5-Hete |  |  |  |
|  | 0.3 | 100 | 8.0 |
|  | 4.0 | 103 | 8.0 |
|  | 40.0 | 101 | 10.0 |
